# Cognitive Impairment in Children of Alcoholics

**Published:** 1995

**Authors:** Robert O. Pihl, Kenneth R. Bruce

**Affiliations:** Robert O. Pihl, Ph.D., is a professor and Kenneth R. Bruce, B.Sc., is a Ph.D. candidate in clinical psychology in the Department of Psychology at McGill University, Montreal, Canada

**Keywords:** cognitive process, children of alcoholics, brain wave, scientific model, behavioral problem, risk factors, predictive factor

## Abstract

Excessive alcohol consumption can be associated with cognitive impairment not only in drinkers but also in their offspring. Studies of children of alcoholics (COA’s), and particularly of sons of male alcoholics (SOMA’s), have identified a characteristic cognitive profile. COA’s frequently have deficits in verbal skills, classification of verbal and visual stimuli, abstract thinking, and goal-directed planning. SOMA’s show additional deficits in visuospatial abilities, perceptual motor skills, and learning and memory. A model is described that explains how the observed cognitive deficits may contribute to the behavioral problems frequently observed in COA’s and to their risk of becoming alcoholic themselves. However, only some, but not all, COA’s exhibit a cognitive profile predictive of behavioral problems.

Between 5 and 10 percent of adults in the United States abuse alcohol or are alcohol dependent as determined by the diagnostic criteria of the *Diagnostic and Statistical Manual of Mental Disorders, Fourth Edition* of the American Psychiatric Association (Grant et al. 1994). The multiple causes underlying alcoholism^1^ are not yet well understood. However, three findings may provide some insight and direction for future research. First, it appears that alcohol dependence runs in some families and can have a genetic component (Cloninger 1987). For example, first-degree relatives of alcoholics, such as children of alcoholics (COA’s; for a more detailed definition, see box), are at least four times more likely to be alcohol dependent than are people with no alcohol-dependent relatives (family history-negative [FHN] people) (Goodwin 1985). Second, among alcoholics, men outnumber women roughly three to one (Grant et al. 1994). And third, males from families with a history of alcoholism are most at risk for becoming alcohol dependent—up to nine times more than are males from families with no history of alcoholism (Cloninger et al. 1981). In light of these findings, the study of COA’s, especially of sons of male alcoholics (SOMA’s), may help determine what predisposes some people to develop drinking problems and may provide more general information on some of the causes and genetic contributions to alcohol abuse and dependence.

**Children of Alcoholics (COA’s)**

This term refers to sons and daughters of male and female alcoholics. Not all studies of COA’s apply the term consistently; some studies use it even though they include only subgroups of COA’s (e.g., only male or female, only with alcoholic mother or father). These inconsistencies make the comparison and summary of COA studies very difficult and may lead to confusing results.

**Sons of Male Alcoholics (SOMA’s)**

This term refers to a specific subgroup of COA’s. The characteristics of SOMA’s can be compared either with COA’s in general (i.e., who have not been subdivided) or with other subgroups, such as daughters of alcoholics or children of female alcoholics.

As described below, one important area to study when determining people’s risk profiles for alcoholism is their cognitive ability. Cognitive abilities include mental operations (e.g., concentrating, learning, memory, and abstract thinking) that are performed to evaluate external events. Skills such as response planning and mental flexibility, which are required to produce appropriate behaviors in response to external events and sensory stimuli, also are considered cognitive abilities. Cognitive functions are required for most everyday activities. For example, a student who is asked a question by a teacher must perform several cognitive functions to respond: The student has to pay attention to the question; understand it; search his or her memory for the appropriate facts or deduce an answer from given facts; formulate and give an answer; and, finally, wait for and assess the teacher’s feedback on the correctness of the answer.

Two main reasons underlie the focus on cognitive abilities when analyzing the risk profile of COA’s. First, cognitive abilities appear to be partly familial (for a discussion, see Hesselbrock et al. 1991) and therefore conceivably could be associated with the familial transmission of alcohol dependence. It is important to note, however, that such an association of cognitive status and alcohol dependence does not presuppose that the cognitive deficits seen in some COA’s are exclusively, or even mainly, genetically determined. Environmental factors, which may be similar for all members of a family, also play an important role in the development of cognitive abilities. Second, cognitive abilities often are good predictors of other behaviors, and some researchers have suggested that deficits in certain cognitive abilities may predate and underlie certain behavioral problems, including alcoholism (Peterson and Pihl 1990).

This article summarizes current knowledge of the cognitive functioning and cognitive deficits in COA’s and, more specifically, in SOMA’s. The article also presents a theoretical model proposing that in some COA’s, specific cognitive deficits (e.g., deficits in classifying and planning) might contribute to the development of alcohol dependence and other behavioral problems. Because the article focuses on cognitive deficits in COA’s that predate problem drinking, the cognitive abilities of COA’s who already are problem drinkers will not be addressed—in these cases, any cognitive deficits also could be a consequence of their alcohol consumption. Similarly, cognitive deficits in COA’s suffering from alcohol-related birth defects will not be discussed.

## How Frequent Are Cognitive Deficits in COA’s?

The exact percentage of COA’s exhibiting cognitive deficits is difficult to estimate from the currently available research data. Instead, scientists state that it is “a significant percentage” of COA’s (Tarter et al. 1990, p. 79). One reason for this lack of specific data is that most studies compare groups of COA’s with groups of FHN’s and do not present the percentages of individual COA’s affected. For the most part, “deficits” in neuropsychological tests only can be evaluated using statistical methods that apply to results obtained with groups of subjects, not with individuals. In many cases, these group-based statistics cannot determine whether a given individual is “deficient” on the test. And even in studies using tests that can measure a specific deficit in individual subjects, estimates of the percentage of COA’s showing this deficit have not been presented.

Estimates of the frequency of cognitive deficits in COA’s are complicated further by the fact that statistical differences between the average performance of COA’s and FHN’s are not found in all tests of cognitive functions. Moreover, even when the same test is used, the results vary among studies.

## Limitations of Studies of Cognitive Deficits in COA’s

To determine whether specific cognitive deficits exist that predate and possibly predict the development of alcohol dependence in some COA’s, researchers ideally would need to study the cognitive profiles and drinking behaviors of a large group of COA’s over a long period of time in a prospective study. A cognitive profile of the COA’s would have to be established (e.g., by giving them a comprehensive battery of cognitive tests) before they begin to consume alcohol, which may have harmful effects on brain functioning (for a review of these effects, see Fals-Stewart et al. 1994). The COA’s then would have to be followed over longer periods of time to compare the cognitive profiles of those who do or do not become alcohol dependent. However, researchers have not yet attempted such a prospective study.

Two kinds of studies indirectly address the issue of cognitive deficits that may predict future alcoholism. The first type of study compares the cognitive abilities of alcohol-dependent COA’s with those of matched nonalcoholic COA’s. Presumably, the alcoholic COA’s, unlike the nonalcoholics, have preexisting cognitive deficits predisposing them to alcohol dependence. However, such studies only have limited relevance, because chronic heavy drinking impairs cognitive functioning. The observed differences or deficits in alcoholic COA’s therefore may be the cause and/or the consequence of their alcohol consumption.

The second kind of study attempts to identify differences in the cognitive abilities of at-risk subjects, such as nonalcoholic COA’s, and matched not-at-risk subjects, such as FHN’s. Cognitive deficits found among COA’s but not among FHN’s could be predictive of future alcohol dependence. However, a lack of difference between COA’s and FHN’s in this type of study is difficult to interpret, because only a minority of COA’s become alcohol dependent; the majority do not^2^ (Chassin et al. 1991; Russell 1990). Thus, the lack of group differences could mean that all COA’s only have small, statistically insignificant cognitive deficits or that only a few COA’s (perhaps those who will later develop alcoholism) have statistically significant deficits.

Inferences about the types of deficits found in COA’s but not in FHN’s can best be made when several studies (such as the ones described above) are summarized. However, even in such an analysis, the conclusions may be ambiguous, because usually groups of COA’s, rather than individuals, are examined. Therefore, even if many studies find a particular deficit in COA’s, it could indicate either a moderate deficit in many of the COA’s studied or it could represent a strong deficit in only a subset of the COA’s in each study.

## Cognitive Deficits in COA’s

Despite the caveats presented above, a vast amount of research has attempted to characterize cognitive deficits of COA’s. The following section focuses on the general findings of four comprehensive research reviews^3^ (Hesselbrock et al. 1991; Pihl et al. 1990*a* ;Sher 1991; Tarter et al. 1990). A summary of the results is presented in table 1.

From the results of the various studies, a cognitive profile of COA’s emerges that includes deficits in verbal skills (including deficits in verbal intelligence^4^ and other more specific verbal abilities^5^), in classification and categorization (i.e., abstract thinking) of both verbal and visual stimuli, and in goal-directed strategic planning. However, the studies detected no consistent deficits in COA’s on measures of learning and memory (Hesselbrock et al. 1991; Sher 1991) and on measures of perceptual and motor skills, including spatial abilities (Hesselbrock et al. 1991; Pihl et al. 1990*a*; Sher 1991). Also, COA’s typically do not show performance deficits on comprehensive standardized neuropsychological test batteries (Hesselbrock et al. 1991; Sher 1991; Tarter et al. 1990).

Despite the general patterns described above, inconsistencies exist among individual studies in the findings of cognitive deficits of COA’s. These inconsistencies may be due in part to differences in subjects used. For example, the subjects’ early use of alcohol, coexisting psychological and medical conditions, and family histories sometimes are documented inadequately. Subjects may be misclassified as COA’s or FHN’s because they either do not know relevant information about their families’ drinking histories or are not forthcoming with it (Schuckit et al. 1995). Subgroups of COA’s—for example, males or females—within risk categories also must be considered. Finally, the selection of subjects may affect study outcome: Recruiting subjects among university students likely excludes COA’s or SOMA’s with severe cognitive deficits (e.g., Sher et al. 1991).

The test measures employed to assess cognitive functions also may contribute to inconsistencies or misinterpretations of study results. Many cognitive/neuropsychological tests assess only very general mental skills. These skills, in turn, require several separate underlying skills, which may not be distinguishable in the general tests. Thus, a subject may have one very specific cognitive deficit—for example, poor concentration skills—which affects his or her responses on a variety of tests that require concentration as well as other abilities. Consequently, the subject appears to have far-ranging deficits when in fact only one skill is impaired. Future studies therefore should assess not only general mental abilities but also more specific cognitive functions.

## Studies of SOMA’s

Because men are more likely than women to become alcohol dependent and because genetic factors appear to affect male COA’s more strongly than female COA’s, most cognitive studies have focused on a subgroup of COA’s, the SOMA’s. However, SOMA’s are not a homogeneous group. They include both men from families in which only the father is alcohol dependent (unigenerational families) and men from families in which several male relatives are alcohol dependent (multigenerational families). Several studies suggest that compared with men from unigenerational families—and possibly with female COA’s—men from multigenerational families have greater cognitive deficits or that a higher percentage of men from multigenerational families have cognitive deficits (Pihl et al. 1990*b*; Tarter et al. 1989; Harden and Pihl 1995). Therefore, it is important to know a SOMA’s family history and genetic background in detail when assessing his cognitive deficits.

Pihl and colleagues (1990*a*) compared the cognitive functioning of SOMA’s, COA’s, and FHN’s (table 2). Groups of SOMA’s consistently differ from matched FHN’s on various aspects of cognitive functioning. Like COA’s in general, SOMA’s have deficient verbal skills, deficient abstracting and planning skills, and lower verbal and performance intelligence scores. However, these deficits may be more prevalent or more severe in SOMA’s—particularly in multigenerational SOMA’s—than in other COA’s (Pihl et al. 1990*b*). In addition, and unlike other COA’s, SOMA’s also have relatively poor visual-spatial abilities and perceptual motor skills and show a variety of learning and memory skill deficits compared with FHN’s.

## Electroencephalographic Brain Wave Studies

A subject’s cognitive abilities can be evaluated not only by using neuropsychological tests but also by measuring electrical brain activity while the subject is at rest or is performing an experimental task. The brain activity is recorded from electrodes placed on many areas of the subject’s scalp, which produce an electroencephalogram (EEG). EEG waves recorded over a period of time reflect the brain activity in the regions under the electrodes, usually the cortex^6^ or other brain centers performing higher cognitive functions. Some of the EEG findings in COA’s and SOMA’s are summarized in table 3.

When the EEG waves of resting subjects are recorded, SOMA’s exhibit an abnormal pattern, with more high-frequency waves than found in FHN’s (Gabrielli et al. 1982). Similarly, male and female COA’s show idiosyncratic brain wave responses when they are intoxicated (Pollock et al. 1983). The implications of these characteristic patterns for the cognitive abilities of COA’s and SOMA’s are unclear at this time.

EEG’s also can record brain activity in response to repeated simple sensory stimuli, such as visual or auditory signals. One EEG wave evoked by such stimuli is called the P300 response, because it occurs roughly 300 milliseconds after the stimulus is presented. P300 is thought to represent the brain responses most typically involved in evaluating the meaning and familiarity of stimuli or events. A quantitative summary, or meta-analysis, of P300 studies of SOMA’s recently was published by Polich and colleagues (1994). The study found that in general, SOMA’s tend to respond less strongly to repeated stimuli (i.e., the amplitude of their P300 wave is lower) than do control subjects. One interpretation is that as a result of this response, SOMA’s may attribute less importance to repeated stimuli in their environment and may easily become bored with repeatedly presented stimuli or with tasks that require prolonged attention (Pihl et al. 1990*a*).

In contrast, when a stimulus is presented for the first time, SOMA’s respond as fast or even faster than do FHN males: The P300 wave is detected earlier and has a higher amplitude in SOMA’s than in FHN’s. This enhanced EEG response is paralleled by, and may be related to, increased cardiovascular responses of SOMA’s to new stimuli or situations (Peterson and Pihl 1990). Taken together, these findings indicate that compared with FHN’s, SOMA’s may respond more strongly to new or unexpected stimuli but may pay less attention to repeated stimuli. These findings from EEG studies, combined with the neuropsychological analyses of cognitive deficits discussed earlier and with results from behavioral observations (see below) led to development of a model—the information-processing model—to explain how cognitive deficits contribute to behavior patterns exhibited by SOMA’s.

## An Information-Processing Model for SOMA’s

Behavioral studies have found that many SOMA’s exhibit externalizing behaviorsuch as impulsivity, attention deficits, out-of-control social behavior, heightened levels of activity, and poor emotional regulation—that is similar to behavior patterns in people with mild dysfunction of the prefrontal cortex (reviewed in Pihl et al. 1990*a*). The prefrontal cortex and other brain areas that transmit signals to and from it are important for abstract classification, planning skills, and behavioral control (e.g., modulating the general arousal level^7^ and maintaining attention). These functions are relevant to performing complex goal-specific activities, recognizing and processing internal and external information, and regulating behavior according to its consequences (see Peterson and Pihl 1990).

The information-processing model proposes that dysfunction in the prefrontal cortex or in the brain areas transmitting signals to and from it could lead to difficulties in the processing of salient (e.g., emotional, new, or threatening) information, which in turn could result in a state of general arousal. This reaction usually ceases when the information is classified correctly and a successful behavioral strategy is implemented. Deficits in both classification and planning would sustain a state of prolonged arousal and leave the person without an appropriate response strategy. Inappropriate responses, in turn, could contribute to the behavioral problems that frequently are observed in SOMA’s.

Preliminary research findings from two laboratories have provided initial support for the notion that deficits in categorization and goal-directed planning play a central role in shaping the cognitive abilities and behavior of SOMA’s, as described below:

- The characteristic P300 patterns suggest that SOMA’s may have difficulties adjusting to and correctly processing external events. This deficit may, in turn, contribute to problems in classifying external stimuli as either familiar or new (see Pihl et al. 1990*b*).
- The poor performance of SOMA’s on tests of memory and abstract classification also implies that they may have difficulties categorizing events as familiar or unfamiliar, meaningful or not.
- Both sober and intoxicated SOMA’s showed poorer performance than did matched FHN controls in a battery of neuropsychological tests designed to evaluate the functioning of the prefrontal cortex and the brain areas transmitting signals to and from it (Peterson et al. 1992).
- Adolescent SOMA’s from multigenerational families performed more poorly than did IQ-matched male FHN’s on four tests sensitive to intact frontal functioning but did not perform more poorly on tests sensitive to the functioning of other brain areas (Harden and Pihl 1995).
- A recent study by Ozkaragoz and Noble (1995) found deficits in memory and planning in SOMA’s with actively alcohol-dependent fathers^8^ compared with IQ-matched FHN’s.

## Implications for “Real World” Problems of SOMA’s

The cognitive deficits found in SOMA’s may help explain some of their problems in everyday situations (Pihl et al. 1990*b*). Deficits in learning and memory skills may contribute to academic problems, which are more prevalent among SOMA’s than among FHN’s and possibly than among other COA’s as well (Pihl et al. 1990*b*). In addition, SOMA’s appear to be more likely to exhibit externalizing behavior than do COA’s. Child and adolescent SOMA’s frequently may be hyperactive and have conduct disorders (Pihl et al. 1990*a*). They tend to be disruptive in the classroom, attend school less consistently, complete fewer school years, and perform less well academically than do their FHN peers. SOMA’s also have been described as impulsive and not considering the consequences of their actions. It seems plausible that these behavioral problems are associated with cognitive patterns, such as deficits in information processing, classification, and planning (Peterson and Pihl 1990). Such a link between a specific cognitive profile and a behavioral pattern is supported by findings that a similar cognitive profile may underlie the externalizing tendency or physical aggression in some adolescent FHN males (Seguin et al. in press).

**Figure 1:**
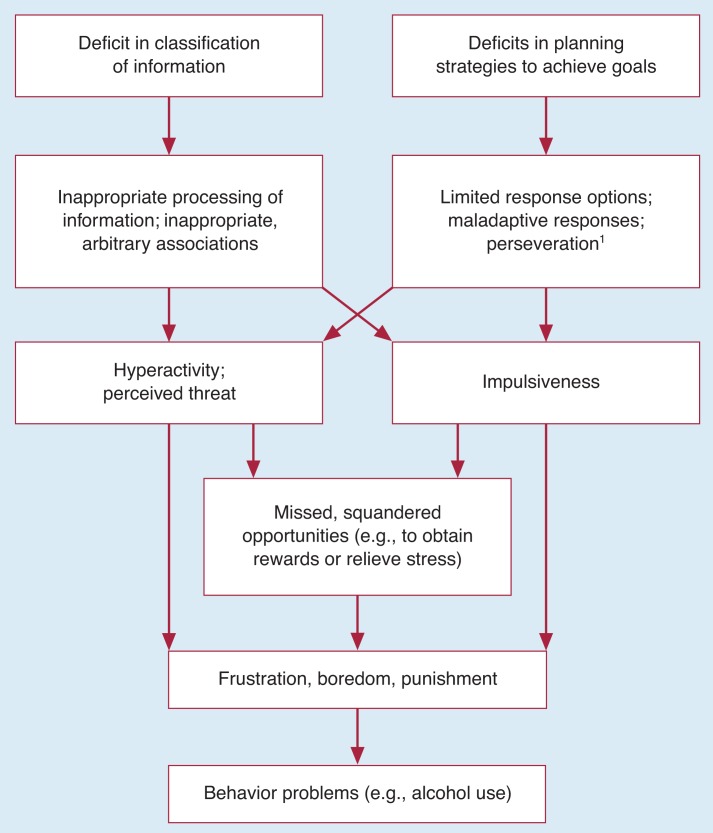
Model of how cognitive deficits in children of alcoholics may lead to behavioral problems. ^1^Perseveration is the excessive repetition of a response.

Impulsivity, which likely is associated with poor planning abilities and the prolonged arousal resulting from misclassified information (as proposed in the information-processing model), could cause SOMA’s to act inappropriately in a given situation. Thus, they might miss or squander opportunities—for example, chances to obtain rewards, relieve stress, or reduce negative emotions—which in turn could lead to frustrating and/or punishing experiences. This model, which is represented in figure 1, can be illustrated best with an example, such as behavior in a classroom setting.

Complex mental functions are required continuously in a classroom setting, for example, in response to a teacher’s question. A SOMA with the cognitive pattern described above may have difficulties understanding new situations and problems or recognizing familiar situations. This may leave the student in a state of heightened arousal, with feelings of apprehension and anxiety. If heightened arousal coincides with poor planning and problem-solving skills, the student’s response to a given problem may be impulsive and ineffective. The resulting poor academic performance may cause the student to miss or squander opportunities for positive experiences (e.g., academic success, positive feedback from peers and adults, or satisfaction with own performance) and eventually may lead to academic marginalization, externalizing behavior, and social problems.

The information-processing theory also offers an explanation as to why SOMA’s are at risk for developing alcohol problems. Acute alcohol intoxication could relieve the anxiety or emotional stress associated with the negative consequences of these information-processing deficits, even if intoxication objectively decreases performance. Consequently, SOMA’s learn that drinking is a reliable way of self-medication and persist in this behavior if left without adequate response alternatives.

## Implications for Identification and Treatment of Cognitive Deficits

Research on interventions to help prevent and remediate cognitive and other problems in COA’s is in its relative infancy. Educational approaches are likely candidates for such interventions (see Williams 1991), but individualized programs for COA’s have yet to be researched. However, the research findings indicating that only some, but not all, COA’s exhibit a cognitive profile that may predict certain life problems, including alcohol dependence, allow several conclusions.

First, all COA’s should not be grouped together in research studies. Doing so will undoubtedly continue to produce confusing and confounding results. Instead, subgroups of COA’s—for example, with respect to gender or family history of alcoholism—should be evaluated separately. Second, to identify cognitive deficits associated with the risk for alcoholism, prospective studies are needed comparing COA’s who become alcoholic with those who do not. Third, it has been theorized, according to the information-processing model, that COA’s with certain cognitive deficits (e.g., classifying and planning) are more likely to develop alcoholism (for more information, see Peterson and Pihl 1990). If further studies support this theory, a helpful step toward primary intervention could be to measure these abilities in preadolescent and adolescent COA’s (Harden and Pihl 1995). Targeted education programs to remediate these deficits in affected COA’s potentially could be a fruitful approach toward prevention of alcoholism and its related problems.

## Summary

A wide range of cognitive deficits can be found in COA’s. In general, COA’s show deficits in verbal abilities and in classifying and planning. In contrast, deficits in learning or visual-motor (including spatial) abilities have not been found consistently. Among COA’s, SOMA’s appear to be most strongly affected by deficits in several aspects of cognitive functioning (e.g., verbal ability, abstract thinking, problem-solving, IQ scores, or learning and memory). The information-processing model presented here explains how these deficits could contribute to the academic and behavioral problems of SOMA’s, including their propensity for alcohol dependence. Future studies should focus on these and other risk characteristics of SOMA’s to develop effective alcoholism prevention, detection, and treatment approaches.

## Figures and Tables

**Table 1:** Cognitive-Neuropsychological Findings in Children of Alcoholics (COA’s)^1^

Cognitive Variable -	Research Findings
Intelligence	
Verbal performance	COA’s have lower scores (Refs. 1–4); male COA’s have lower scores (Ref. 3) COA’s with multiple male alcoholic relatives have lower scores than COA’s with only an alcoholic father (Ref. 1)
Verbal skills	Adopted COA’s have poorer ability (Ref. 1) COA’s have poorer general verbal ability (Refs.1–4)
Memory	
Verbal	Most studies suggest no impairment in COA’s (Refs. 2,4, but cf. Ref. 3) COA’s with multiple male alcoholic relatives have lower scores than COA’s with only an alcoholic father (Ref. 1)
Nonverbal	Most studies suggest no impairment in COA’s (Refs. 2,4)
Perceptual and Motor Skills (including spatial skills)	COA’s with multiple male alcoholic relatives have poorer skills than COA’s with only an alcoholic father (Ref. 1) Some inconsistent data showing deficits in COA’s (Refs. 2, 3), but much evidence against these deficits (Ref. 4) Some evidence for increased body sway while standing for COA’s; the findings are inconsistent, possibly due to procedural and sampling differences (Ref. 2) Most studies suggest no differences between intoxicated COA’s and family history-negative (FHN) subjects on body sway (Ref. 2) Sober COA’s are not impaired on other sensorimotor domains (Refs. 2,4) but may differ from FHN’s while intoxicated (Ref. 2)
Standard Neuropsychological Test Batteries	Little evidence for consistent deficits in COA’s (Refs. 1,2,4) Some evidence for statistical differences between COA’s and FHN’s but small and not clinically meaningful (Ref. 2)
Abstracting and Planning	Adopted COA’s have poorer ability (Ref. 1) Alcoholic COA’s (vs. FHN alcoholics) have poorer ability (Refs. 1,3,4) Generally, COA-FHN differences are relatively small but are present (Refs. 2–4)

1 The findings are presented for COA’s compared with family history-negative subjects except where indicated.

SOURCES: References (1) Tarter et al. 1990; (2) Sher 1991; (3) Pihl et al. 1990*a*; (4) Hesselbrock et al. 1991.

**Table 2:** Cognitive Characteristics of Sons of Male Alcoholics (SOMA’s)

Compared with family history-negative (FHN) subjects, SOMA’s show consistently poor performance in the following areas: Performance on tests of linguistic ability Categorization and grouping (abstract thinking) abilities Problem-solving skills Full-scale (total) IQ scores Performance (visual-perceptual) IQ scores^1^
Compared with FHN’s and children of alcoholics in general, SOMA’s show consistently poor performance in the following areas: Learning and memory skills Visual-spatial abilities Perceptual-motor skills

1 The performance IQ score is a measure derived from five separate tests of perceptual organization, including nonverbal reasoning skills, the ability to employ visual images in thinking, and the ability to process visual and spatial information.

**Table 3:** Electroencephalographic (EEG) Findings in Children of Alcoholics (COA’s)^1^

Variable	Research Findings
Resting Brain Wave Activity	Excess of EEG activity indicating heightened arousal in male COA’s (Ref. 1, but cf. Ref. 2) Increased amounts of certain brain waves in intoxicated COA’s compared with intoxicated family history-negative subjects (Ref. 1)
Brain Wave Responses to Stimuli	Delayed (Refs.1–3) and dampened (Refs.1–4) P300 response in male COA’s to complex (but not to simple) familiar events.

1 The findings are presented for COA’s as compared with family history-negative subjects except where indicated.

SOURCES: References (1) Tarter et al. 1990; (2) Sher 1991; (3) Pihl et al. 1990*a*; (4) Polich et al. 1994.

## Footnotes

1 Throughout the remainder of this article, the terms “alcohol dependence,” “alcoholism,” or “problem drinking” are used interchangeably. However, the definitions for these terms vary among studies of children of alcoholics. Some studies have used the definitions of the American Psychiatric Association; others have used standardized tests to diagnose alcohol dependence. This divergence in definitions and terms may contribute to some of the inconsistencies in the research findings.

2 Estimates for the prevalence of alcohol dependence among COA’s range from about 5 percent to about 50 percent (Chassin et al. 1991; Russell 1990).

3 Each review was based on a minimum of 20 relevant primary studies from a variety of research groups. For more information, readers are referred to these reviews and to the studies on which they are based.

4 Verbal intelligence refers to measures such as vocabulary, mental arithmetic ability, short-term verbal memory, detecting similarities between two objects, and common sense reasoning in everyday situations.

5 These abilities include general skills with words or language, such as reading and auditory comprehension, verbal expression, and performance on tests in which word responses are required.

6 For a definition of this and other technical terms, refer to the central glossary on p. 136–137.

7 “Arousal” refers to a state of agitation with feelings of nervousness or anxiety, apprehension, and hyperreactivity.

8 Some scientists postulate that sons of actively alcohol-dependent fathers, as opposed to abstinent alcohol-dependent fathers, are at a greater genetic risk for becoming alcohol dependent themselves (Ozkaragoz and Noble 1995).
